# CHILDHOOD VITILIGO: RESPONSE TO METHYLPREDNISOLONE ORAL MINIPULSE THERAPY AND TOPICAL FLUTICASONE COMBINATION

**DOI:** 10.4103/0019-5154.53185

**Published:** 2009

**Authors:** Imran Majid, Qazi Masood, Iffat Hassan, Dilshad Khan, Muzammil Chisti

**Affiliations:** *From Department of Dermatology, STD and Leprosy, Govt. Medical College, Srinagar, Kashmir, India*

**Keywords:** *Childhood vitiligo*, *methylprednisolone*, *oral minipulse*

## Abstract

**Background:**

Childhood vitiligo is always a challenge to treat, especially when the disease is progressing rapidly in such a patient. Oral minipulse with betamethasone has been tried in childhood vitiligo and also in some other immune mediated skin disorders with good results.

**Aims:**

The aim of the present study was to see the overall efficacy of methylprednisolone oral minipulse therapy in combination with topical fluticasone in progressive childhood vitiligo. The combination was tried to achieve a significant amount of repigmentation of vitiligo lesions already present at the initial visit.

**Materials and Methods:**

Four hundred children with progressive vitiligo were enrolled for this study and were prescribed oral methylprednisolone on two consecutive days every week in a minipulse form for a period of six months. In addition, the patients were instructed to apply fluticasone ointment topically once a day on their vitiligo lesions. The patients were assessed for the remission achieved as well as the extent of repigmentation of their already existent lesions.

**Results:**

More than 90% of patients went into complete remission after the start of the therapy. Moreover, about 65% (two-thirds) of patients achieved good to excellent repigmentation of lesions at the end of six months of therapy. The therapy was also well tolerated and the side effects seen were almost negligible.

**Conclusions:**

Oral minipulse treatment with methylprednisolone is an effective treatment option for controlling the disease spread in childhood vitiligo and with the addition of topical fluticasone the extent of repigmentation achieved is also quite significant.

## Introduction

Vitiligo is one of the commonest skin disorders, affecting about 1% of the world population.[1] The disease presents more commonly in children than in adults. Treatment options for progressive vitiligo are quite limited, especially in pediatric patients.[2] This stems from the fact that systemic treatments commonly used for the disease have the potential of causing serious adverse effects in this age group.[2] Both oral as well as topical corticosteroids have been used as treatment options in vitiligo with variable therapeutic results. Oral steroids, if given as a daily therapy in children, can cause serious side effects, especially HPA axis suppression and growth retardation due to premature epiphyseal closure. Different pulse therapies with systemic steroids have been devised to minimize these side effects, of which the oral minipulse (OMP) therapy has been used particularly in vitiligo,[3–5] and more recently, in alopecia areata and lichen planus.[6–8]

## Materials and Methods

Four hundred patients of progressive vitiligo from ethnic Kashmiri origin and belonging to the pediatric age group (below 15 years of age) were enrolled for this study. Patients with any of the morphological variants of vitiligo, but having a progressive disease were included. The epidemiological data from all the patients were noted down and this included weight and height of the patients, morphological type of vitiligo, age of onset, duration of vitiligo, any familial involvement, percentage of body surface area involved, predominant sites on the body involved, as well as the past treatments used for the disease. A thorough clinical assessment was made to exclude other related cutaneous and systemic disorders. Laboratory investigations for other related disorders were however performed only if there was a clinical evidence of such a possibility. After this thorough assessment, the patients were initiated on OMP therapy of methylprednisolone for two consecutive days every week up to a minimum duration of six months. The dose of methylprednisolone used was 0.8mg/kg body weight in each patient with the maximum dose of 32 mg on each day. This was also combined with once daily topical application of 0.01% fluticasone ointment to the lesional skin at bedtime. The patients were monitored every two weeks for the first month and then once a month for the rest of the study period. The efficacy of treatment was judged on two counts: An arrest of the disease process as well as the extent of repigmentation of lesions already present. The treatment was continued for a minimum duration of two months in every patient and thereafter in only those patients who showed remission of the disease. Patients showing a continuous progression of the disease, even after two months of treatment from the time of initiation were administered alternative therapies. Any adverse reactions to the therapy were also noted down, in the form of weight gain (comparison of initial weight with that at the end of therapy), Cushingoid facies, growth retardation, gastrointestinal upset, bone pains, and acne. The extent of repigmentation achieved was graded as poor (<25%), good (25-50%), very good (50-90%), and excellent (>90%). In those patients in whom the disease had gone into remission and who had a partial repigmentation of lesions at the end of six months study period, the OMP treatment was continued and either topical PUVASOL or any other topical treatment was added to the treatment protocol. However, the final assessment of treatment for the study was done only at the end of six months.

## Results

The age of patients enrolled for the present study ranged from a minimum of 18 months to a maximum of 15 years (chosen as the upper age limit). The age distribution of patients is shown in Table 1.

**Table 1:** Age and sex distribution of study group patients

Age group (years)	Males	Females	Total
<2	1	3	4
2-5	12	34	46
6-10	65	112	177
11-15	56	117	173
Total	134	266	400

Females outnumbered males in every age group and the overall male:female ratio was about 1:2 [Table 1].

The duration of vitiligo ranged from a minimum of about one week to a maximum of four-and-a-half years with a mean of 4.3 months.

The different morphological types of vitiligo recorded were vitiligo vulgaris including focal vitiligo (272 patients), segmental vitiligo (83 patients), acrofacial vitiligo (34 patients), and mucosal vitiligo (11 patients).

Family history of vitiligo was recorded in 87 patients accounting for 21.75% of the whole study group.

Alopecia areata was the commonest coexistent skin disorder seen in 22 patients (5.5%), followed by psoriasis in 17 patients (4.25%). No significant association was found with any systemic disorder, except hypothyroidism that was recorded in five patients (1.25%). Other disorders seen in the study group are enumerated in Table 2.

**Table 2:** Associated disorders seen in the study population

Associated disorder	No. of patients	Percentage
Alopecia areata	22	5.5
Psoriasis	17	4.25
Pityriasis alba	13	3.25
Discoid eczema	3	0.75
Chronic urticaria	3	0.75
Hypothyroidism	5	1.25

### Response to therapy

Overall, 57 patients out of the study population of 400 did not come for regular follow up and were dropped from the assessment protocol. Out of the remaining 343 patients who completed the study, there were significantly only 33 (9.6%) patients who continued to have fresh lesions of vitiligo even at the end of two months of the minipulse treatment. The other group of 310 patients, which constituted 90.4% of the follow-up population, went into complete remission as far as the progression of vitiligo was concerned. Another significant observation was that almost all these patients, at least, remained in remission for the whole study period of six months. Patients who continued to have a progressive disease even after two months from therapy initiation were dropped from any further assessment and were given alternative treatment options.

The extent of repigmentation of lesions was noted down at every follow-up visit and the total repigmentation achieved at the end of treatment was also recorded. In about one-third of patients (107) the extent of pigmentation was poor (<25% repigmentation), while in the rest two-thirds of patients the repigmentation was graded as good to excellent [Table 3]. In 41 patients (13.2%) the lesions resolved completely within the six months study period (100% repigmentation) and they did not require any further therapy for their vitiligo.

**Table 3:** Percentage repigmentation seen with therapy after a maximum of six months

Repigmentation of lesions	Poor (<25% pigmentation)	Good (25-50% pigmentation)	very good (50-90% pigmentation)	Excellent (>90% pigmentation)
Number of patients	107	92	70	41
Percentage	34.5	29.7	22.6	13.2

Of the different morphological forms of vitiligo the percentage of patients going into remission was almost similar with every group. However, as far as the repigmentation of lesions was concerned, patients with vitiligo vulgaris and segmental vitiligo were seen to respond better than those with acrofacial or mucosal vitiligo.

The extent of repigmentation achieved was also correlated with the duration of vitiligo and it was observed that the response was inversely proportional to the duration of vitiligo [Table 4]. Patients having vitiligo of less than three months duration were seen to fare much better than those with a disease of a relatively longer duration.

**Table 4:** Correlation of repigmentation of lesions with duration of disease

Duration of vitiligo	Patients showing arrest of disease	Patients showing good to excellent repigmentation	Percentage response
<3 months	131	117	89.3
3-6 months	82	54	65.8
6 months to 2 years	46	26	56.5
>2 years	51	6	11.7

Adverse effects that were noted with the therapy were a transient weight gain in 57 patients, gastric irritation in 18 patients, tinea capitis and/or corporis in 16 patients, and precipitation of acne in 11 patients. No skin atrophy or any other local adverse effects at the lesional sites because of topical steroid use could be demonstrated.

## Discussion

Treating a patient of vitiligo is always a difficult task and the job becomes even more challenging when the patient is a child and his/her disease is progressing at a fast rate. This is due to the fact that the treatment options available for this group of patients are quite limited, not universally effective, and liable to cause troublesome adverse effects. Treatment options like immunosuppressants, oral psoralens,[9] and even conventional doses of oral steroids cannot be employed in children with vitiligo because of the side effect profile of these drugs. Steroids have always been a treatment option in vitiligo, both in topical as well as oral forms. However, oral steroids have proved to be double-edged weapons in vitiligo, and when given to a child, they can lead to irreversible and serious side effects like premature closure of epiphysis leading to stunted growth, Cushing's syndrome, etc. A modified method of prescribing oral steroids, which is claimed to minimize their side effects to a large extent is the OMP therapy.[4–6] Here a relatively large dose of an oral steroid is given on two consecutive days per week with a rest period of five days in between the doses. In the original studies that have commented upon the use of OMP in vitiligo, the steroid used has been either betamethasone or dexamethasone.[3–8] The drug has been given in a dose of 5mg or approximately 0.1mg/kg body weight in these studies. The dosage has however not been too fixed in these studies with frequent increases made in the dose in unresponsive patients. We have modified this approach as we have used a relatively safer steroid, methylprednisolone, instead of betamethasone, in a fixed dose of 0.8mg/kg body weight. This does is comparable in potency with the dose of betamethasone used in the previous studies.

An additional modification in the present study was the addition of topical fluticasone ointment 0.01% once daily to the treatment regimen. This was done with the aim of increasing the chances of repigmentation of lesions already present as the original studies of OMP therapy in vitiligo have failed to demonstrate any significant repigmentation in their enrolled patients.[4–5] Moreover, the patient or his/her attendant is always keen to apply something on the white patches as it gives them a sense of participation in the patient's treatment. This modification, in fact, went a long way in improving the follow up in our patients.

The response to the treatment schedule described in the present study can be termed as quite encouraging on an overall basis. We had a response rate of >90% if the halting of progression of vitiligo is taken as the criterion for judging the response to therapy. This no doubt is a remarkable figure in itself, as the first thing a patient or his/her parent and even the treating physician want to see is a halt in spread of the disease in case of progressive vitiligo. As far as the pigmentation of already present lesions is concerned the response was good to excellent in about two-thirds (65.5%) of patients [Table 4], while about one-third of patients showed a poor response (< 25% pigmentation). Another very important observation was that 41 patients in our series, comprising about 12% of the responders had complete (100%) repigmentation of all their vitiligo lesions and they did not require any other therapy for their disease.

Spontaneous remission and repigmentation is known to occur in vitiligo patients, but the remission rate of 90% that was achieved with the OMP treatment described in this study cannot be ascribed to spontaneous remission alone. Further, repigmentation in vitiligo has been observed with the use of topical steroids,[10] especially clobetasol propionate,[11] betamethasone valerate, and even with new molecules like fluticasone[12] and mometasone. However, the repigmentation got with these molecules has been modest in most of the studies. We thus conclude that combination of OMP with methylprednisolone and topical fluticasone ointment is a safe and effective treatment option in the management of progressive childhood vitiligo.
